# The influence of seasonal factors on the incidence of peritoneal dialysis-associated peritonitis

**DOI:** 10.1080/0886022X.2020.1804401

**Published:** 2020-08-11

**Authors:** Ying Zeng, Xiaomei Jiang, Sheng Feng, Linsen Jiang, Zhi Wang, Huaying Shen, Shan Jiang

**Affiliations:** Department of Nephrology, the Second Affiliated Hospital of Soochow University, Suzhou, P.R. China

**Keywords:** Peritoneal dialysis, peritonitis, seasonal variation, temperature, humidity

## Abstract

**Objectives:**

To investigate the effects of climatic variables on peritoneal dialysis-associated peritonitis (PDAP) among patients receiving PD, such as seasonal variations in temperature and humidity.

**Methods:**

A retrospective analysis was performed on PD patients, from 1 January 2011, to 31 December 2019. We evaluated the influence of seasonal factors on peritonitis rates and outcomes.

**Results:**

Over the 9-year study period, 667 peritonitis episodes occurred, in 401 PD patients. Diarrhea-associated peritonitis occurred more frequently in summer compared with other seasons. Eating raw and cold food was identified as the primary cause of peritonitis in the summer. More peritonitis episodes occurred during summer. The peritonitis rate associated with gram-negative bacteria (*p* = 0.050) during summer was higher than those in all other seasons. The gram-negative bacterial peritonitis rate was positively correlated with monthly mean temperature (*r* = 0.504, *p* < 0.01) and humidity (*r* = 0.561, *p* < 0.01). A similar trend was observed for Enterobacterial peritonitis (temperature: *r* = 0.518, *p* < 0.01; humidity: *r* = 0.456, *p* = 0.001). Logistic regression analysis showed that summer was a risk factor for peritonitis (*p* = 0.041). Peritonitis prognosis during summer was significantly worse than those for all other seasons (*p* = 0.037).

**Conclusions:**

Seasonal variations exist in the incidence of dialysis-associated peritonitis, with peak incidents caused by gram-negative bacteria in the summer. High average temperature and humidity are associated with significant increases in the gram-negative bacteria and Enterobacterial peritonitis rates. Peritonitis prognosis during summer is worse.

## 1. Introduction

Peritoneal dialysis-associated peritonitis (PDAP) is the primary complication of peritoneal dialysis (PD) treatment is a common cause for the failure of the peritoneal dialysis technique and patient death [1,2]. Recently, the incidence of peritonitis has decreased significantly, associated with the improvements in the peritoneal dialysis connection system, the accumulation of experience, and the emphasis on education [3,4]; however, large differences in the incidence of peritonitis have been identified among different regions and at various centers [5–7]. Many factors, such as the patient’s immune status and age, adherence to aseptic techniques, operation techniques, education, environment, and changes in the spectrum of bacteria responsible for PDAP, can affect the occurrence of peritonitis [8]. Among these factors, seasonal factors are often ignored. Seasonal variations may affect the incidence of peritonitis by changing the health of patients, the infection method, the distribution of normal skin microbiota, and the microenvironment in which microorganisms can survive and thrive [8]. However, few studies have examined this aspect. Therefore, we retrospectively analyzed the clinical data available for PDAP patients treated at our center, from 1 January 2011, to 31 December 2019. To determine effective prevention strategies to reduce infection, we explored the impacts of seasonal variations on the incidence of PDAP.

## 2. Materials and methods

### 2.1. Case selection

All episodes of PD-related peritonitis that occurred at the peritoneal dialysis center of the Second Affiliated Hospital of Suzhou University, from 1 January 2011, to 31 December 2019, were reviewed. Dialysis methods included intermittent PD (IPD) and continuous ambulatory PD (CAPD). Patients with IPD and CAPD were changed 2–5 times a day and received 2 L each time. The dialysis prescription was 4–10 L, daily.

During the study period, 667 episodes of peritonitis were recorded, and the case records were reviewed. Patient demographic information, peritonitis occurrence details, such as the onset time, inducement, and clinical symptoms, the results of the most recent laboratory examinations before peritonitis, the microbiology results, therapeutic responses, and clinical outcomes were examined.

### 2.2. Diagnosis and treatment of peritonitis

Peritonitis was diagnosed according to the International Society of Peritoneal Dialysis (ISPD) guidelines, established in 2016 [9], with at least two of the following three items required for peritonitis diagnosis. (1) The presence of peritonitis symptoms and signs, such as abdominal pain and a cloudy dialysate effluent, either with or without fever. (2) A white blood cell count (WBC) in the dialysate effluent of more than 100 × 10^6^/L, of which neutrophil counts represented greater than 50%. (3) The identification of a pathogenic microorganism, by staining or culturing the dialysate effluent. Relapse infections, as defined by the ISPD guidelines, were counted as one single episode, whereas recurrent and repeat infections were counted as separate episodes.

The management of PD-related peritonitis, at our center, involved empirical, anti-infective treatments. Antimicrobial therapies utilized first-generation treatments of cephalosporin or vancomycin, combined with a third-generation cephalosporin treatment or aminoglycoside drugs, administered intraperitoneally. The antibiotics were adjusted, according to the results of the dialysate effluent culture and drug sensitivity tests.

### 2.3. The definition of peritonitis prognosis

Peritonitis clinical outcomes were divided into cure and failure. A cure was defined as a WBC count less than 100 × 10^6^/L in the dialysate effluent and a negative culture results after antibiotic treatment. Treatment failure included the conversion to permanent hemodialysis and death related to peritonitis. Death related to peritonitis was defined as the death of a patient, due to peritonitis, during hospitalization for peritonitis, or within 4 weeks of peritonitis [9].

### 2.4. Seasonal variations

The patients with PDAP were divided into four groups, according to the four seasons, and data regarding the weather was obtained for each season: spring (from March to May), summer (from June to August), autumn (from September to November), and winter (from December to February). Using daily average temperature and humidity data for Suzhou, from 2011 to 2019, provided by the China Meteorological data network, we further calculated the monthly average temperatures and humidities for all seasons across all years of our study.

### 2.5. Statistical analysis

Statistical analysis was performed using SPSS 22.0. Patients were grouped according to years, months, seasons, and pathogens, and the incidence of PDAP was compared among the various groups. Continuous variables that conformed to normal distributions were expressed as the mean ± standard deviation, whereas categorical variables with normal distributions were expressed as numbers and percentages. Peritonitis rates were calculated according to the standardized recommendations established by the ISPD [9]. All episodes of peritonitis that occurred while patients were receiving PD were divided by the number of days that patients were receiving PD, during the evaluated time period (month, season, year, or study period). PD occurrence was expressed as episodes per patient-year at risk, with a 95% confidence interval (CI). A one-way analysis of variance (ANOVA) was used to compare continuous data and the Chi-square test was used to compare categorical data, among multiple groups. Correlations between pathogenic microorganisms and monthly average temperature and humidity were further explored, using Pearson’s correlation coefficient. Factors that influenced peritonitis were screened by univariate and multivariate logistic regression analyses. Temperature and humidity data were processed using square root to obtain better linear results during the Pearson’s correlation coefficient test. For all comparisons, *p* < 0.05 was deemed significant.

## 3. Results

### 3.1. Population characteristics

From 2011 to 2019, 909 PD patients were admitted to our center, all of whom were permanent residents in Suzhou. During this period, 607 episodes of peritonitis occurred, in 401 patients, including 192 males (47.88%) and 209 females (52.12%), with an average age of 61.72 ± 16.43 years. Primary diseases included chronic glomerulonephritis, in 231 cases (57.61%), diabetic nephropathy, in 72 cases (17.96%), hypertensive nephropathy, in 34 cases (8.48%), lupus nephritis, in 7 cases (1.75%), polycystic kidney, in 10 cases (2.49%), purpura nephritis, in 5 cases (1.25%), obstructive nephropathy, in 6 cases (1.50%), gouty nephropathy, in 5 cases (1.25%), and unknown nephropathy, in 31 cases (7.73%).

A total of 308 of 909 PD patients used drugs to prevent exit-site infections: among 92 cases (29.87%) treated with gentamicin, 13 cases (14.13%) developed peritonitis; among 24 cases (7.8%) treated with mupirocin, 3 cases (12.5%) developed peritonitis; among 192 cases (62.3%) that received iodophor or concentrated sodium treatment, 47 cases (24.5%) developed peritonitis. No significant difference in the incidence of peritonitis was observed among the three infection-prevention treatment groups (*p* = 0.078).

Patients with peritonitis were divided into four groups, according to the season during which the peritonitis case occurred. The results showed no significant differences among the groups (*p* > 0.05, Table 1).

**Table 1.** Characteristics of all PD patients with peritonitis during the study period, from 2011 to 2019.

Characteristics	Spring (*n* = 160)	Summer (*n* = 186)	Autumn (*n* = 167)	Winter (*n* = 154)	*p-*Value
Age (years)	63.13 ± 16.57	61.38 ± 17.11	63.99 ± 14.78	58.87 ± 16.32	0.253
Male (*n*, %)	72 (45.0)	92(49.5)	74 (44.3)	78 (50.6)	0.759
PD duration (months)	38.13 ± 26.54	42.62 ± 19.11	39.83 ± 24.91	30.79 ± 24.65	0.372
BMI (kg/m^2^)	22.41 ± 3.20	23.26 ± 3.51	22.73 ± 2.84	22.63 ± 3.56	0.878
Potassium (mmol/L)	3.73 ± 0.67	3.67 ± 0.91	3.64 ± 0.66	3.56 ± 0.57	0.275
Hemoglobin (g/L)	107.77 ± 24.18	109.20 ± 23.61	108.13 ± 21.95	109.43 ± 18.34	0.246
Albumin (g/L)	32.12 ± 5.21	31.78 ± 6.34	32.10 ± 5.72	34.14 ± 4.79	0.384
Phosphorus (mmol/L)	1.40 ± 0.52	1.43 ± 0.54	1.47 ± 0.49	1.46 ± 0.44	0.193
Total KT/Vurea	1.68 ± 0.37	1.69 ± 0.48	1.76 ± 0.41	1.81 ± 0.40	0.103
Total weekly Ccr ml/min/1.73 m^2^	53.21 ± 15.19	52.18 ± 18.10	53.29 ± 14.78	56.82 ± 16.96	0.590

BMI: Body mass index; KT/Vurea: dialysis clearance of urea; Ccr: creatinine clearance rate.

The clinical manifestations and predisposing factors associated with each peritonitis case were compared. The results showed that diarrhea symptoms occurred significantly more frequently during summer than during the other seasons (*p* = 0.042). Eating raw and cold foods was the primary cause of peritonitis during summer (*p* = 0.086), which had marginal statistical significance. Details regarding these factors can be found in Table 2.

**Table 2.** Comparisons of the clinical manifestations and predisposing factors for patients during different seasons.

Outcome	Spring (*n* = 160)	Summer (*n* = 186)	Autumn (*n* = 167)	Winter (*n* = 154)	*p*-Value
Clinical manifestations [*n* (%)]					
Fever	47 (29.4)	73 (39.2)	65 (38.9)	51 (33.1)	0.172
Abdominal pain	142 (88.8)	160 (86.0)	146 (87.4)	123 (79.9)	0.118
Diarrhea	40 (25.0)	66 (35.5)	38 (22.8)	42 (27.3)	0.042
The amount of ultrafiltration decreased	105 (65.6)	117 (62.9))	130 (70.7)	101 (65.6)	0.488
Muddy dialysate	137 (85.6)	164 (88.2)	144 (86.2)	126 (81.8)	0.417
Cause [*n* (%)]					
Operation	53 (33.1)	59 (31.7)	64 (38.3)	42 (27.3)	0.207
Eating raw and cold food	48 (30.0)	63 (33.9)	37 (22.2)	40 (26.0)	0.086
Constipation	15 (9.4)	27 (11.3)	17 (10.2)	24 (15.6)	0.319
Systemic infection	16 (10.0)	15 (8.1)	18 (10.8)	22 (14.3)	0.318
Exit-site infection	13 (8.1)	13 (7.0)	16 (9.6)	12 (7.8)	0.845
Malnutrition	6 (3.8)	5 (2.7)	7 (4.2)	9 (5.8)	0.530
Other	9 (5.6)	4 ( 2.2)	8 (4.8)	5 (3.2)	0.455

### 3.2. Overall seasonal variations in the peritonitis rate

**Figure 1.**
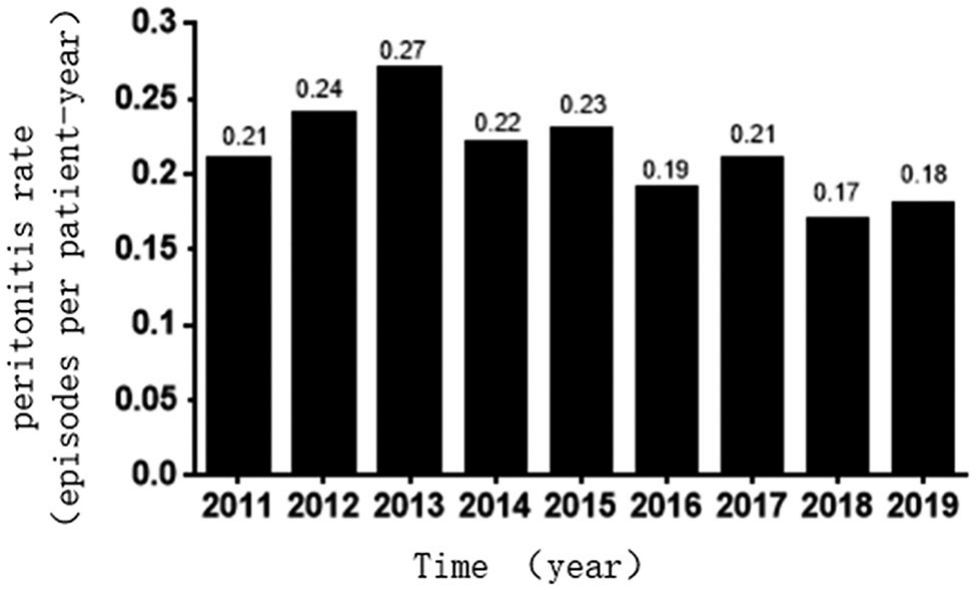
Changes in the peritonitis rate at the Second Affiliated Hospital of Suzhou University, from 2011 to 2019.

The peritonitis rate between 2011 and 2019 are summarized in Figure 1. The average incidence of PDAP between 2011 and 2019 was 0.21 episodes per patient-year (i.e., 1 episode/70.66 patient-months). From 2014 to 2019, the infection rate began to show a downward trend and maintained a low level.

From 2011 to 2019, the average temperatures for Suzhou were 16.6 °C during spring, 27.9 °C during summer, 19.2 °C during autumn, and 5.9 °C during winter. Among 667 episodes of peritonitis, 186 episodes occurred during summer, 167 during autumn, 160 during spring, and 154 during winter. A trend toward seasonal variations in the peritonitis rate was observed, with more peritonitis episodes occurring during the summer (0.24 episodes per patient-year), which was the hottest season, followed by spring and autumn (0.21 episodes per patient-year), and the lowest numbers of peritonitis episodes occurring during winter (0.19 episodes per patient-year) when the climate was cool and dry. Since 2011, except for 2012, 2015, and 2016, the incidence of PDAP was higher during the summer than during the other three seasons. However, no significant differences were observed in the average peritonitis rates among the seasonal groups during these 9 years (*p* = 0.423), as shown in Table 3.

**Table 3.** Overall peritonitis rate for different seasons (episodes/patient-year).

Year	Spring	Summer	Autumn	Winter
2011	0.15	0.28	0.23	0.24
2012	0.27	0.25	0.20	0.25
2013	0.25	0.30	0.26	0.23
2014	0.14	0.29	0.25	0.25
2015	0.28	0.25	0.21	0.18
2016	0.21	0.22	0.25	0.15
2017	0.25	0.26	0.19	0.17
2018	0.14	0.24	0.20	0.18
2019	0.18	0.26	0.21	0.12

**Figure 2.**
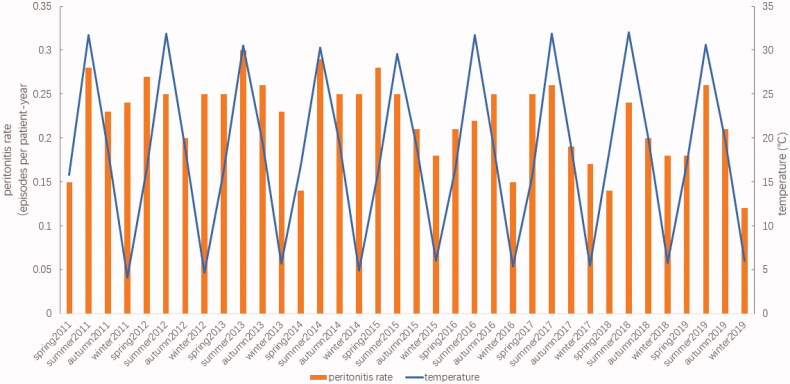
The incidence of peritonitis and varying temperatures during different seasons, from 2011 to 2019.

**Figure 3.**
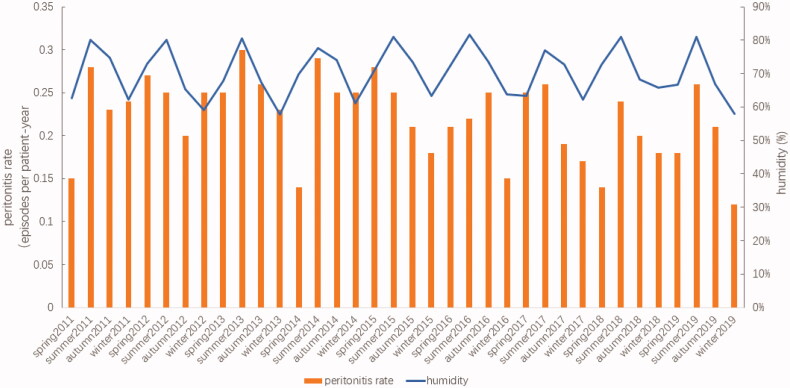
Incidence of peritonitis and humidity changes during different seasons, from 2011 to 2019.

The relationships between the incidence of peritonitis and the average temperature and humidity, for spring, summer, autumn, and winter, between 2011 and 2019, are shown in Figures 2 and 3.

### 3.3. Seasonal variations in microbiologic causes

Of the 667 identified episodes of peritonitis, 658 episodes (98.65%) were cultured, using the dialysate effluent, among which, 566 were successfully isolated and identified, representing a positive rate of 86.02%. The microbiologic causes these peritonitis episodes are summarized in Table 4. Enteric bacteria includes both gram-negative Enterobacterales and Enterococcus.

**Table 4.** Microbiologic causes of peritonitis.

Organisms identified	Case [*n* (%)]
Gram-positive organisms	326 (49.54)
Coagulase-negative *Staphylococci*	137 (20.82)
*Streptococci*	104 (15.81)
*Enterococcus*	26 (3.96)
*Staphylococcus aureus*	21 (3.19)
Gram-negative organisms	182 (27.66)
Enterobacterial	208 (31.61)
*Escherichia coli*	76 (11.55)
*K. pneumoniae*	35 (5.32)
Fungus	20 (3.04)
Culture-negative	92 (13.98)
Others	38 (5.78)
Total	658

The incidence of peritonitis caused by individual bacterial species was further analyzed, summarized in Table 5. Significant seasonal variations were observed for the incidence of peritonitis caused by gram-negative bacteria, which was the highest during the summer (*p* = 0.050). Meanwhile, the incidence of PDAP caused by coagulase-negative *Staphylococcus*, *Staphylococcus aureus*, and *Enterobacteria* during the summer was higher than those for other seasons; however, this result did not reach significance.

**Table 5.** Relationships between microorganisms and seasonal variations.

Organisms identified	Spring	summer	Autumn	Winter	*p-*Value
Gram-positive organisms	0.108	0.093	0.088	0.117	0.626
	(0.079–0.137)	(0.070–0.117)	(0.066–0.111)	(0.094–0.140)	
Coagulase-negative Staphylococci	0.054	0.058	0.042	0.053	0.292
	(0.032–0.075)	(0.042–0.074)	(0.029–0.055)	(0.038–0.068)	
Streptococci	0.047	0.023	0.030	0.038	0.453
	(0.031–0.062)	(0.014–0.031)	(0.018–0.043)	(0.026–0.050)	
Staphylococcus aureus	0.003	0.011	0.006	0.007	0.220
	(0.001–0.006)	(0.004–0.018)	(0.001–0.012)	(0.001–0.014)	
Gram-negative organisms	0.048	0.091	0.056	0.043	0.050
	(0.030–0.066)	(0.069–0.113)	(0.041–0.071))	(0.029–0.058)	
Enterobacterial	0.052	0.095	0.061	0.050	0.113
	(0.035–0.070)	(0.074–0.117)	(0.043–0.078)	(0.033–0.068)	
Culture-negative	0.029	0.033	0.042	0.032	0.570
	(0.016–0.042)	(0.020–0.047)	(0.026–0.058)	(0.019–0.044)	
Fungus	0.005	0.006	0.010	0.006	0.747
	(0.000–0.009)	(0.000–0.012)	(0.001–0.019)	(0.001–0.013)	

### 3.4. Relationships with temperature and humidity

**Figure 4.**
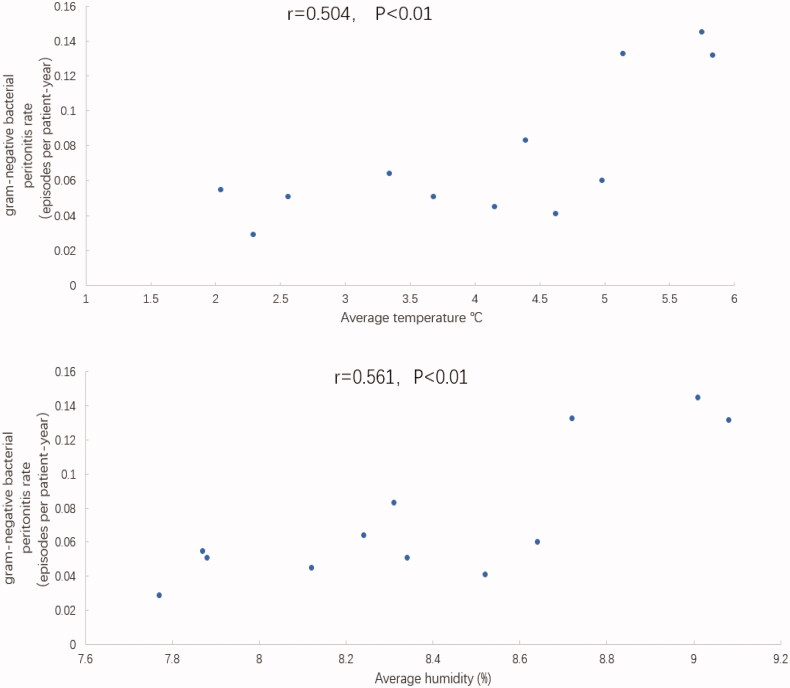
Relationships between the gram-negative bacterial peritonitis rate and average temperature and average humidity.

**Figure 5.**
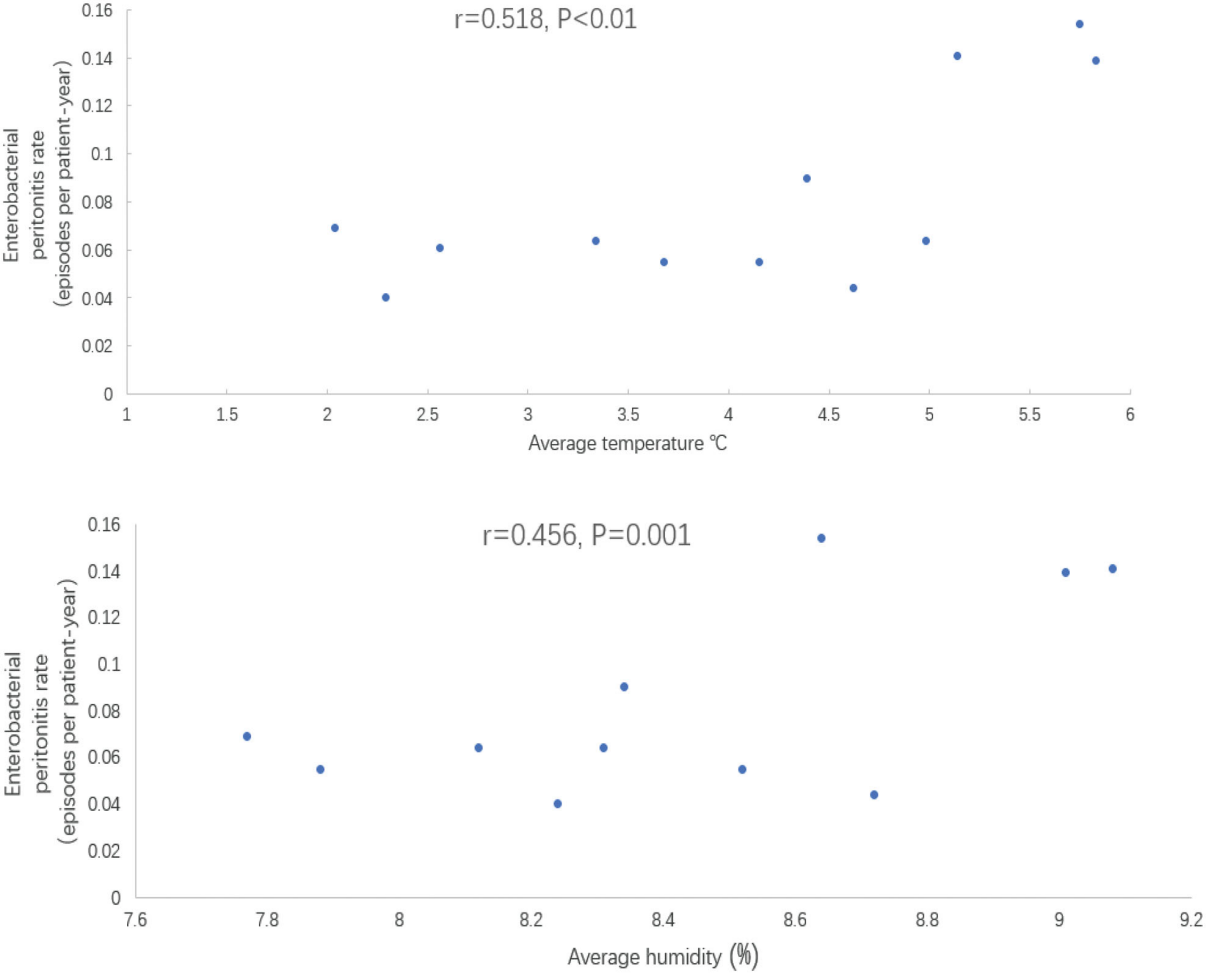
Relationships between the enterobacterial peritonitis rate and the average temperature and average humidity.

Pearson’s correlation coefficient analysis was used to analyze the correlation between PDAP incidents associated with various pathogens and the monthly average temperature and humidity, which were treated by square root. The results showed a significant correlation between monthly average temperature and the incidence rate for gram-negative bacterial peritonitis (Pearson’s *r* = 0.504, *p* < 0.01), and monthly average humidity was also positively correlated with the gram-negative bacterial peritonitis incidence rate (Pearson’s *r* = 0.561, *p* < 0.01), as shown in Figure 4. In contrast, a modest correlation was observed between the monthly average temperature and the incidence rate of Enterobacterial peritonitis (Pearson’s *r* = 0.518, *p* < 0.01). The monthly average humidity was also positively correlated with the Enterobacterial peritonitis rate (Pearson’s *r* = 0.456, *p* = 0.001), as shown in Figure 5.

### 3.5. Influencing factors for peritonitis

Using peritonitis as the dependent variable, the following variables were included as covariates, to establish a binary logistic regression model: age, gender, diabetes mellitus, hemoglobin, albumin, serum creatinine, cholesterol, potassium, c-reactive protein (CRP), and season. Because the lowest incidence of peritonitis occurred during winter, the winter group was treated as the reference group. First, a logistic univariate regression analysis was performed, and the results showed that age, anemia, decreased serum albumin level, elevated serum CRP levels, and summer were independent risk factors for peritonitis (*p* < 0.05, Table 6).

**Table 6.** Logistic univariate regression analysis for peritonitis.

Variable	*B* value	OR value	95% CI	*p*-Value
Age	1.245	3.472	1.687–8.587	0.000
Gender	−0.212	0.809	0.654–1.002	0.052
Diabetes mellitus	0.405	1.499	0.587–3.833	0.397
Hemoglobin (g/L)	−0.012	0.988	0.635–0.981	0.022
Albumin (g/L)	−0.751	0.472	0.851–0.996	0.007
Serum creatinine	−0.213	0.808	0.430–1.517	0.507
Cholesterol	0.428	1.534	0.92–1.409	0.327
Potassium (mmol/L)	0.574	1.775	0.667–3.517	0.411
hs-CRP(Log_10_) (mg/L)	1.685	5.392	3.673–7.917	0.000
Seasonal grouping				
Winter	Reference	Reference	Reference	Reference
Spring	0.604	1.830	0.706–4.744	0.214
Summer	0.934	2.545	1.430–4.528	0.041
Autumn	0.695	2.003	0.806–2.942	0.109

All factors with *p* < 0.1 from the univariate regression analysis were included in the logistic multivariate regression analysis. The results showed that age, decreased serum albumin level, and increased serum CRP levels remained independent risk factors for peritonitis (*p* < 0.05, Table 7) in the multivariate analysis.

**Table 7.** Logistic multivariate regression analysis for peritonitis.

Variable	*B* value	OR value	95% CI	*p*-Value
Age	0.307	1.360	1.005–1.837	0.012
Gender	−0.019	0.981	0.958–1.006	0.134
Hemoglobin (g/L)	−0.022	0.978	0.945–1.013	0.220
Albumin (g/L)	−0.577	0.561	0.483–0.925	0.034
hs-CRP(Log_10_) (mg/L)	0.721	2.056	1.202–3.591	0.002
Seasonal grouping				
Winter	Reference	Reference	Reference	Reference
Spring	0.048	1.049	0.975–3.130	0.401
Summer	0.307	1.359	0.985–4.637	0.246
Autumn	0.026	1.026	0.603–3.899	0.383

### 3.6. Seasonal variations peritonitis outcomes

Among the 667 recorded episodes of PDAP, 582 episodes were cured, with a total cure rate of 87.3%. Treatment failure outcomes included the conversion to permanent hemodialysis and death associated with peritonitis. A total of 85 episodes ended in failure, including 52 patients of permanent hemodialysis transfer and 33 patients of death. The prognosis of peritonitis was compared across the four seasons. The results showed that during summer, 34 episodes ended in treatment failure, which accounted for the highest proportion (40%) among the four seasons, including 23 patients transferred to hemodialysis, and 11 patients of death. The peritonitis treatment failure was the highest during the summer (18.3%), which represented a significant difference (*p* = 0.037, Table 8).

**Table 8.** Clinical outcomes of peritonitis, according to the four seasons [(Case ％)].

Outcome	Spring	Summer	Autumn	Winter	*p*-Value
Cure	147 (91.8%)	152 (81.7%)	148 (88.6%)	135 (87.7%)	0.037
failure	13 (8.2%)	34 (18.3%)	19 (11.4%)	19 (12.3%)	
hemodialysis	9 (5.6%)	23 (12.4%)	11 (6.6%)	9 (5.8%)	
death	4 (2.6%)	11 (5.9%)	8 (4.8%)	10 (6.5%)	

## 4. Discussion

In this retrospective study, we found that the average incidence of PDAP was 0.21 episodes per patient-year, between 2011 and 2019, which was far lower than the 0.50 episodes per patient-year reported by the 2016 ISPD guidelines for peritonitis [9]. For the most recent 5-year period, from 2014 to 2019, the peritonitis rate remained low. The occurrence rate, in 2018, was 0.17 episodes per patient-year and, in 2019, was 0.18 episodes per patient-year. These low rates may be due to the continuously strengthened management measures implemented at our center. For example, in the study nearly 34% of patients in our center used antibiotics to prevent exit-sit infection. Continuously improve the experience accumulation of peritoneal dialysis doctors and full-time nurses [10]. Patients are trained for 5–7 days by nurses’ operation explanation, watching videos, guiding patients to operate by themselves and home guidance manual [11]. After the training, oral questions, written examinations and operation examinations are carried out on aseptic operation principle, peritoneal dialysis operation and exit-site dressing change. Regular small lectures are given before the outpatient follow-up, and the reeducation is strengthened through the interaction of cases and questions. The patients with peritonitis, catheter infection, long hospital stay, and interrupted peritoneal dialysis are retrained in aseptic concept, operation technology, exit nursing, infection control and drug compliance [12]. The patients with recurrent peritonitis are visited at home to evaluate the operation technology and home environment, and targeted retraining is conducted in time when problems are found [13]. 24-h hotline and SMS platform are used to guide and deal with emergencies. Regular review for peritoneal dialysis patients, especially difficult, critical and death cases, and timely intervention when problems are found [14].

Although the incidence of peritonitis at our center and other centers in China remains stable and low, large differences exist among Asia, Europe, and America [5–7]. Some scholars believe that regional differences may affect the incidence of peritonitis, and seasonal variations may be one potential risk factor associated with peritonitis [8]. However, few studies have examined this aspect, and the results of existing studies have varied. Kim et al. [8], in South Korea, analyzed the relationship between seasons and PDAP occurrence. The results showed that the highest incidence of PDAP occurred in July, whereas the lowest incidence was recorded for November. The monthly frequency of PDAP was significantly and positively correlated with temperature and humidity. Our study found that the incidence of PDAP during the summer was higher than those during other seasons, reaching 0.24 episodes per patient-year, which is consistent with the conclusions reached by Zhao et al. [15]. This result further supports our view that the incidence of PDAP peaked during the hot and humid months. In a large, multi-center study, conducted from 2003 to 2008, including 6610 Australian peritoneal dialysis patients, the results showed that relative to the occurrence of PDAP during winter, the peritonitis incidence rate ratios for summer, autumn, and spring were 1.02, 1.01, and 0.99, respectively [16]. Based on these findings, the incidence of peritonitis during the summer did not increase significantly, which is different from our results. However, differences among seasons may be less well-defined in tropical countries, such as Australia. The single- vs multicenter nature of each study may also affect the results because differences in climate may be masked by multicenter studies that include centers in different locations of a country, which may have different climates.

However, seasonal variations have been reported to have no significant effects on the occurrence of PDAP associated with specific microorganisms [17, 18]. Therefore, our study analyzed the causative microorganisms associated with PDAP cases. The results showed that the incidence of gram-negative bacterial peritonitis during the summer was significantly higher than those for the other three seasons. Cho et al. [16] also found that significant seasonal variations were observed among the rates of peritonitis caused by gram-negative organisms (with summer and autumn peaks). Gram-negative bacteria associated with peritonitis include *Escherichia coli* and *Klebsiella pneumoniae*, which primarily cause peritonitis through intraluminal infections or blood-borne pathways [19]. Al Hasan et al. [20] found a 35% increase in the infection rate (IR) associated with *E. coli* bloodstream infections (BSIs) during the warmest 4 months of the year (June through September), compared with the IR for the remainder of the year, and a 7% increase the *E. coli* BSI IR was observed for each 5.5 °C increase in average temperature. *In vitro* examinations of the temperature requirements for *E. coli* growth have shown that the *E. coli* doubling rate increases with increasing temperatures until an optimal growth temperature of 35–36 °C is reached [21]. A report from four continents demonstrated that the *in vitro* temperature requirements for the growth of both *E. coli* and *K. pneumoniae* are relatively uniform; therefore, the growth patterns of these two organisms are thought to be similar [22]. Some environmentally acquired bacteria, such as *Acinetobacter* and *Pseudomonas aeruginosa*, will also grow faster with increasing temperatures, and their virulence will be greatly enhanced [16]. Szeto et al. [23] found that humidity is an important environmental factor for *Acinetobacter*-associated peritonitis. These studies support our findings that the incidence of peritonitis caused by gram-negative bacteria peaked during the hot and humid summer period and that the incidence rate was directly proportional to increases in temperature and humidity.

Enteric bacteria are widely distributed throughout the human intestinal system and can primarily be divided into Enterobacterales and Enterococcus spp. Most Enterobacterales are *Escherichia coli* and *Klebsiella pneumoniae* [24–26], whereas the Enterococcus spp are primarily *Enterococcus faecalis* and *Enterococcus faecium* [27]. This study analyzed the relationships between enteric bacterial peritonitis and the seasons. The results showed that the incidence of enteric peritonitis (EP) increased during the summer compared with other seasons; however, no statistical differences in the incidence rate were identified, which may be related to the small sample size. We also found that increased temperature and humidity were associated with the increased incidence of EP. As early as 1950, Schweinburg et al. [28] confirmed that EP-causing bacteria can pass through the intestinal wall and enter the abdominal cavity, by using radioactive element-labeled *Escherichia coli*. Szeto et al. [24] also confirmed that EP is primarily associated with GI diseases. In cases associated with incomplete intestinal walls, the excessive proliferation of intestinal bacteria, and weakened host peritoneal defense functions, normal parasitic bacteria in the intestinal tract can enter the abdominal cavity, either through the lymphatic system or directly through the intestinal wall, due to the weakened barrier. Based on our analysis of the causes associated with peritonitis, we found that the primary cause of peritonitis during the summer was eating raw and cold foods and diarrhea symptoms, compared with the causes for other seasons. However, Elshafie et al. [29] believed that diarrhea caused by *Campylobacter* may be related to PD-related EP. Therefore, we speculate that EP is directly connected to summer patients being more likely to consume raw, cold, or unclean foods, which more easily cause intestinal infections, diarrhea, and other symptoms. Moreover, the temperature and humidity during the summer are higher than those during other months, and intestinal bacteria multiply excessively, making infections passed through the intestinal cavity more likely. Vomiting, diarrhea, and the use of potassium-free peritoneal dialysis can often lead to hypokalemia in PD patients. Hypokalemia is associated with malnutrition, which weakens the defense function of the peritoneum against pathogenic bacteria [30]. Enteric bacteria in hypokalemia patients with peritoneal dialysis proliferate excessively, which destroys the normal microenvironment of the intestinal tract [31]. Additionally, the lack of potassium in the blood can weaken intestinal peristalsis, which can lead to intestinal paralysis. Enteric bacteria can easily move across the intestinal wall to the abdominal cavity, causing abdominal infections.

We did not observe differences in the occurrence of gram-positive bacterial peritonitis associated with seasonal variations, which may be related to the widely used peritoneal dialysis connection system [23]. However, the rates of peritonitis caused by specific microorganisms were associated with seasonal variations. We found that the incidence of PDAP associated with coagulase-negative *Staphylococcus* and *Staphylococcus aureus* during the summer was higher than those during other seasons. Peritonitis caused by *Staphylococcus* is closely related to contaminations that occur during operation because *Staphylococcus* is commonly found on the skin and mucous membranes of the human body. Hot and humid climates generally favor the accumulation of sweat and dirt around the catheter exit site, which may promote the growth of *Staphylococcus* in the catheter and the outlet, which may prolong the colonization time [30]. If the operation is not standardized, *Staphylococcus* may increase the risks of peritonitis transmitted through skin or environmental contamination, such as the contamination at the junction, the outlet of the peritoneal dialysis tube, or the loss or loosening of the iodophor cap at the end of the tube [32].

In the univariate regression analysis, we also found that summer was a risk factor for peritonitis. Compared with the other three seasons, the prognosis for PD patients during summer was worse than those during other seasons. Since the center was established, most cases of peritonitis have been treated in a hospital setting. Treatment failures during the summer occurred more often, which may be associated with a higher incidence of EP during the summer. Dong et al. [33] found that compared with gram-positive bacterial peritonitis, gram-negative bacterial peritonitis was associated with more severe clinical symptoms, often accompanied by fever or obvious abdominal pain, with a high hospitalization rate, a high technical failure rate, a high catheter removal rate, and poor prognosis. Huang et al. [34] studied the prognosis of 599 peritonitis episodes and found that the cure rate associated with gram-positive bacteria was significantly higher than that for gram-negative bacteria. The poor prognosis observed for the EP group may be related to the high proportion of gram-negative bacteria-associated cases. All of these findings suggested that the prognosis of peritonitis during the summer is relatively poor and the treatment is more difficult; therefore, we should pay additional attention to these issues.

This study had some limitations. This study was a single-center retrospective study, with a relatively small sample size, and the results may not apply to other centers. Season is a complex factor, including different temperature, humidity and different eating habits. These factors are related to the occurrence of peritonitis. If some confounding factors are corrected, the results may be uncertain. In the follow-up study, we will perform a multi-center, large-sample, prospective study to verify our findings. If we can further confirm the relationship and characteristics associated with seasonal variations of PDAP incidence, these findings may have additional clinical value.

In conclusion, some correlations were identified between the occurrence of PDAP and the seasons. The incidence of PDAP caused by gram-negative bacteria during the summer was higher than those during other seasons. We also found that enteric bacteria were positively correlated with both the monthly average temperature and humidity. During the summer, the cure rate for PDAP was relatively low, associated with poor prognosis. Our findings highlighted the importance of climatic factors on the incidence of PD-related peritonitis. Because hot and, more importantly, humid environments are disadvantageous, maintaining a cool and dry living environment (e.g., using fans, air-conditioners, and dehumidifiers) may help to reduce the incidence of peritonitis. For gram-negative bacterial peritonitis, special attention should be paid to training and education regarding food hygiene and the prevention of intestinal infections. To reduce the incidence of gram-positive bacterial peritonitis, we must emphasize the training of PD patients’ standard operation techniques. We hope to provide effective strategies for the clinical prevention and treatment of PDAP, by counteracting seasonal factors, to reduce the occurrence of peritonitis and improve the prognosis of PD patients.
